# Machine learning, medical diagnosis, and biomedical engineering research - commentary

**DOI:** 10.1186/1475-925X-13-94

**Published:** 2014-07-05

**Authors:** Kenneth R Foster, Robert Koprowski, Joseph D Skufca

**Affiliations:** 1 Department of Bioengineering, University of Pennsylvania, Philadelphia, PA 19104, USA; 2 Department of Biomedical Computer Systems, University of Silesia, Faculty of Computer Science and Materials Science, Institute of Computer Science, ul.Będzińska 39, Sosnowiec 41-200, Poland; 3 Department of Mathematics & Computer Science, Clarkson University, Box 5815, Potsdam, NY 13699-5815, USA

**Keywords:** Artificial intelligence, Classifiers, Image processing, Machine learning, Support vector machine

## Abstract

A large number of papers are appearing in the biomedical engineering literature that describe the use of machine learning techniques to develop classifiers for detection or diagnosis of disease. However, the usefulness of this approach in developing clinically validated diagnostic techniques so far has been limited and the methods are prone to overfitting and other problems which may not be immediately apparent to the investigators. This commentary is intended to help sensitize investigators as well as readers and reviewers of papers to some potential pitfalls in the development of classifiers, and suggests steps that researchers can take to help avoid these problems. Building classifiers should be viewed not simply as an add-on statistical analysis, but as part and parcel of the experimental process. Validation of classifiers for diagnostic applications should be considered as part of a much larger process of establishing the clinical validity of the diagnostic technique.

## Introduction

In recent years, there has been a dramatic increase in the use of computation-intensive methods to analyze biomedical signals. The general approach falls under the rubrics of artificial intelligence or machine learning, in which a computer program “learns” important features of a dataset to enable the user to make predictions about other data that were not part of the original training set. One of many applications of this approach is to create classifiers that can separate subjects into (usually) two or (rarely) more classes based on attributes measured in each subject. An obvious potential use of such a classifier is to analyze biomedical data and detect or diagnose disease.

This commentary focuses on use of support vector machines (SVMs), a computationally intensive statistical technique that emerged as a research topic in the late 1990s, but similar comments would apply to other machine learning techniques as well. The literature on these topics is immense: a search on Google Scholar using keywords SVM and image analysis results in 93,000 cites, on SVM and facial recognition finds 32,000 references, and SVM and speech recognition uncovers 28,000 cites. (Not all of the cites are equally relevant however, since Google Scholar casts a very wide net). Google Scholar finds several thousand recent articles in response search terms SVMs and biomedicine. In the single area of electrocardiogram (ECG) analysis, these include papers on heartbeat detection, arrhythmia classification, diagnosis of heart valve disease, and recognition of sleep apnea from the ECGs.

The wide popularity of machine learning arises in part from the availability of commercial software packages such as the Statistical Toolbox of Matlab (Mathworks, Natick MA USA) or Statistica (Statsoft, Tulsa OK USA) that make it easy for investigators to apply, or misapply, these sophisticated statistical techniques. These make it easy to do quick studies – one can simply download ECG or other data from individuals with various medical conditions from the Internet, subject it to wavelet or other analysis, input the parameters into the software, and then propose a diagnostic technique using the resulting classifier. Investigators who use such software are not necessarily trained or inclined to perform the additional analysis needed to demonstrate the validity or usefulness of their results.

Another problem is the “black box” character of many of these methods, SVM in particular, which makes them prone to false discovery (i.e. finding spurious associations) [1]. Used carelessly, they can create classifiers that appear to perform impressively well– when applied to the original training set –but are useless when applied to new data. Examples are given in cases discussed below.

This present commentary is a result of extensive discussions among members of the editorial board of this journal and other experts about how editors and referees should evaluate papers in biomedical engineering that employ such methods. It is not intended as a scholarly contribution to the voluminous and sophisticated literature in this area e.g. [2-9] but rather to illustrate some major pitfalls of the technique and sensitize readers and reviewers of biomedical engineering papers of issues to be alert for.

### Developing and validating classifiers

Developing a classifier using SVMs or other classification technique consists of several steps: (a) choosing a method of analysis; (b) choosing a set of features or attributes that will be used to classify the subjects; (c) training the classifier; (d) validating the classifier; and (e) evaluating potential errors in the classification [10]. Each step presents opportunities to introduce bias and error into the process.

### Choice and number of attributes

The number and choice of attributes is critical to the success of a classifier. Too many attributes relative to the number of “events” (e.g. sick individuals) leads to overfitting, a result of the classifier learning the data instead of the trend that underlie the data [11]. An analogous problem arises when one fits data to a high order polynomial: if the order of the polynomial is too large relative to the number of data points, a good fit will be obtained but the polynomial will not capture underlying trends in the data and have no predictive value for new data.

As a rule of thumb, more than 10 “events” are needed for each attribute to result in a classifier with reasonable predictive value [12]. Ideally, similar numbers of “healthy” and “unhealthy” subjects would be used in a training set, resulting in a training set that is more than 20 times the number of attributes. Since biomedical engineering studies typically involve a small number of subjects, and there are essentially unlimited numbers of parameters that can be used to characterize biomedical signals, reviewers and readers should be on the lookout for the possibility of overfitting.

Another crucial issue is the choice of attributes [13-15]. For a diagnostic application, the attributes must have some bearing on the disease. Physicians typically interpret physiological data such as an ECG by examining features that are considered to represent medically significant phenomena, for example observing the polarity of certain waves in the ECG.

By contrast, biomedical engineers often train classifiers using abstract characteristics of a signal, for example entropy within a signal or wavelet coefficients. The classifier would be useful only if the attributes captured a significant amount of medically relevant information, and ideally should be independent and not contain confounding variables. Establishing that a set of abstract coefficients is suitable for developing diagnostic applications is just the beginning of the larger clinical validation process, which would normally require extensive clinical trials.

### Validation of predictive model

Validating a classifier involves testing it on a set of subjects (the test set) that is independent of the training set. When the dataset is large, one can simply divide it into a training and test set (hold-out method). An effective and statistically justified validation method that can be used with smaller datasets is leave-one-out cross-validation. In this approach, one sample is removed from the training set, the classifier is recalculated using the remaining training set, and then applied to the holdout sample as a test. This process is repeated in turn for each member of the training set. Other validation methods can be used instead that are computationally less intensive, and some machine learning techniques combine training and validation of classifiers in one process [16,17]. But however it is done, the classifier cannot be validated using the same data that were used to develop the classifier in the first place, which would introduce circularity.

“Independence” is a theoretical construct that impacts on the external validity of the model. For a project involving ECG analysis, different ECG records from the same patient would probably not be sufficiently independent, even if the records were from different days. For developing a medical diagnostic technique, one may need more than individuals selected from a single cohort of subjects (for example, patients of the same physician) if the test is to be used in different medical centers by different physicians.

The quality of the biomedical engineering literature on these topics is extremely varied. At the low end of the quality scale, one can find many papers that report no validation studies at all, but merely show that the classifier works well on the training set, which tells nothing about the predictive value of the classifier when faced with new data. Many other papers lack sufficiently clear description of the validation methods to enable readers to judge the validity of the work. Reviewers of papers need to be sure that enough information is provided to allow them to judge the scientific validity of the validation process used in the study.

### Illustrations of pitfalls in using classifiers

Three following examples illustrate these pitfalls as related to the use of SVM. The first uses a classifier trained on a synthetic dataset consisting of random numbers, while the remaining examples employ a real dataset consisting of ultrasound images from patients with Hashimoto’s inflammation, an autoimmune disease affecting the thyroid, and healthy controls [18].

### Random dataset

In this example, synthetic training sets were created with equal numbers of “healthy” and “ill” individuals. Each individual was assigned 10 attributes consisting of random numbers. Because the attributes were chosen independently from the assigned class, they contain no predictive information. Consequently the accuracy of the classifier when applied to a validation set with equal numbers of “healthy” and “ill” subjects would be 50% due to chance alone.

**Figure 1:**
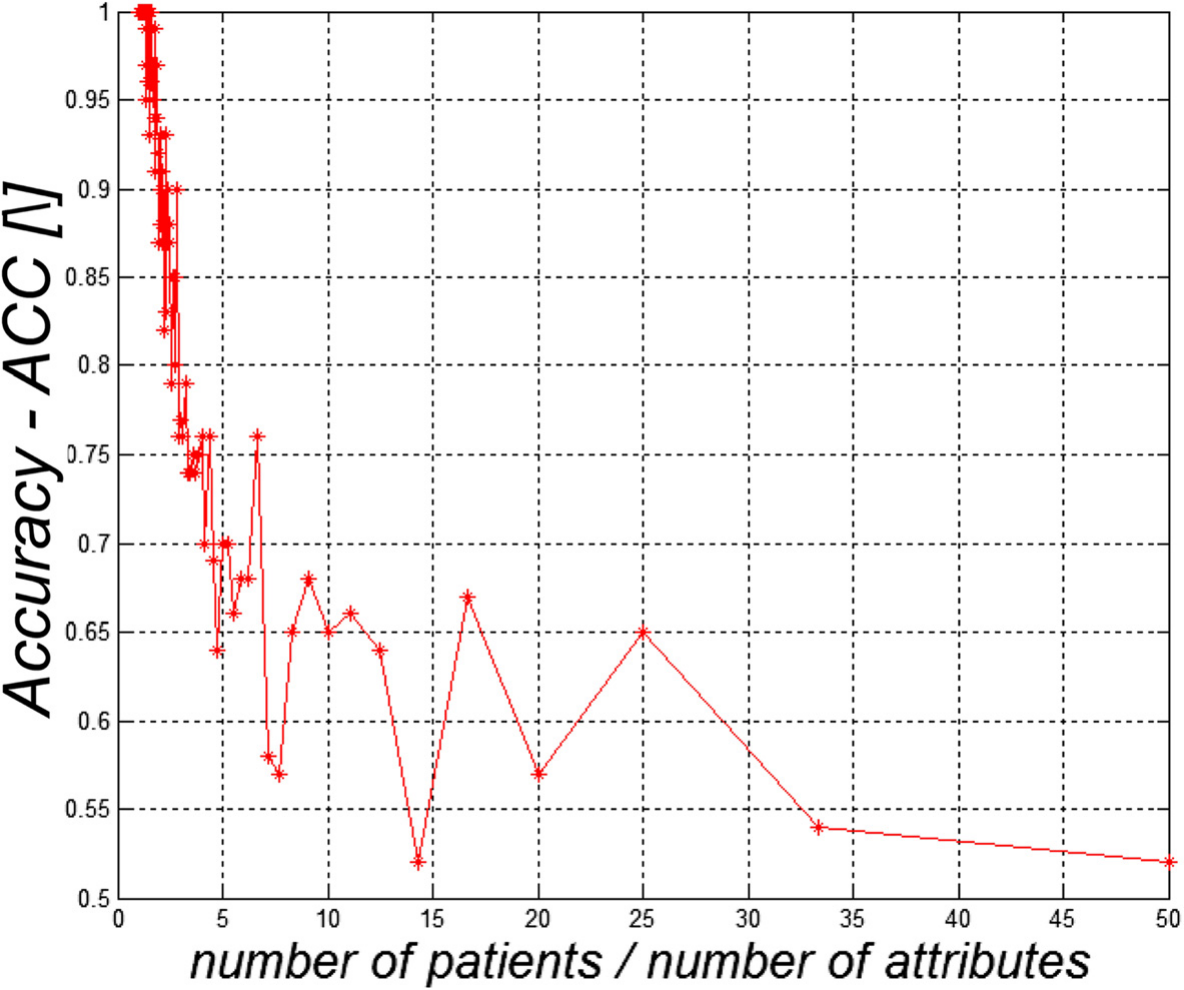
**Apparent accuracy of classifiers ( *ACC *) applied to synthetic training sets of equal numbers of “healthy” and “ill” subjects, with 10 attributes for each subject created using a random number generator.** The horizontal axis is the ratio of the number of “ill” subjects to number of attributes (10 in each case). The increase in accuracy (*ACC* in the vertical axis) for smaller training sets is a result of use of a too-small training set, coupled with post-hoc theorizing. Since the set had an equal number of “patients” and “healthy” individuals, the accuracy of the classifier should be 50% as expected by chance.

Each synthetic training set was used to train a classifier using a linear SVM (Matlab Statistics Toolbox, Mathworks, Natick MA). Figure 1 shows the results of applying the classifier to the *same* training set that had been used to develop it, for training sets of different size.For small training sets (4–6 subjects per attribute, including patients and controls) the classifier seemed to approach 100% in accuracy in Figure 1 due to the circular nature of this (clearly invalid) training/validation. The actual predictive value of the classifier is, of course, nil. Readers should note the remarkably large training/validation set, perhaps 200 “patients” in this case, for the actual level of performance of the classifier to become apparent.

### Clinical ultrasound images – hashimoto’s disease

Data were obtained by one of the present authors (R.K.) in connection with a previous study that examined feature selection as related to the accuracy of a classifier in diagnosing Hashimoto’s inflammation of the thyroid based on ultrasound images [19]. The images of patients were obtained by Dr. W. Zieleźnik and his team [18], and the medical status of each subject had been confirmed by a physician based on the ultrasound images together with other clinical data. In the following example, we consider 250 images from Hashimoto’s patients and the same number from healthy individuals; each image is from a different subject.

Ten attributes were defined for each image: average image power spectrum, regional minimum value on the image, smoothness of the image, the minimum value of brightness of the image, the position of the center of GLCM (Gray-Level Co-occurrence Matrix) matrix gravity, range of contrast in different values of GLCM, and three parameters obtained from square-tree decomposition of the image (for details see [18]). Images were analyzed using custom written software (Matlab, The Mathworks, Natick MA). For purposes of this example, a classifier was trained using a SVM with a linear kernel implemented with Matlab’s Statistics Toolbox.

a. Size of training set.

We investigate the effect of the size of training sets of different size, consisting of images from Hashimoto’s patients and an equal number of healthy controls. In each case, the validation set consisted of 100 images from subjects (50 healthy, 50 ill) that had been held out as a test set (and not used in the training set).

**Figure 2:**
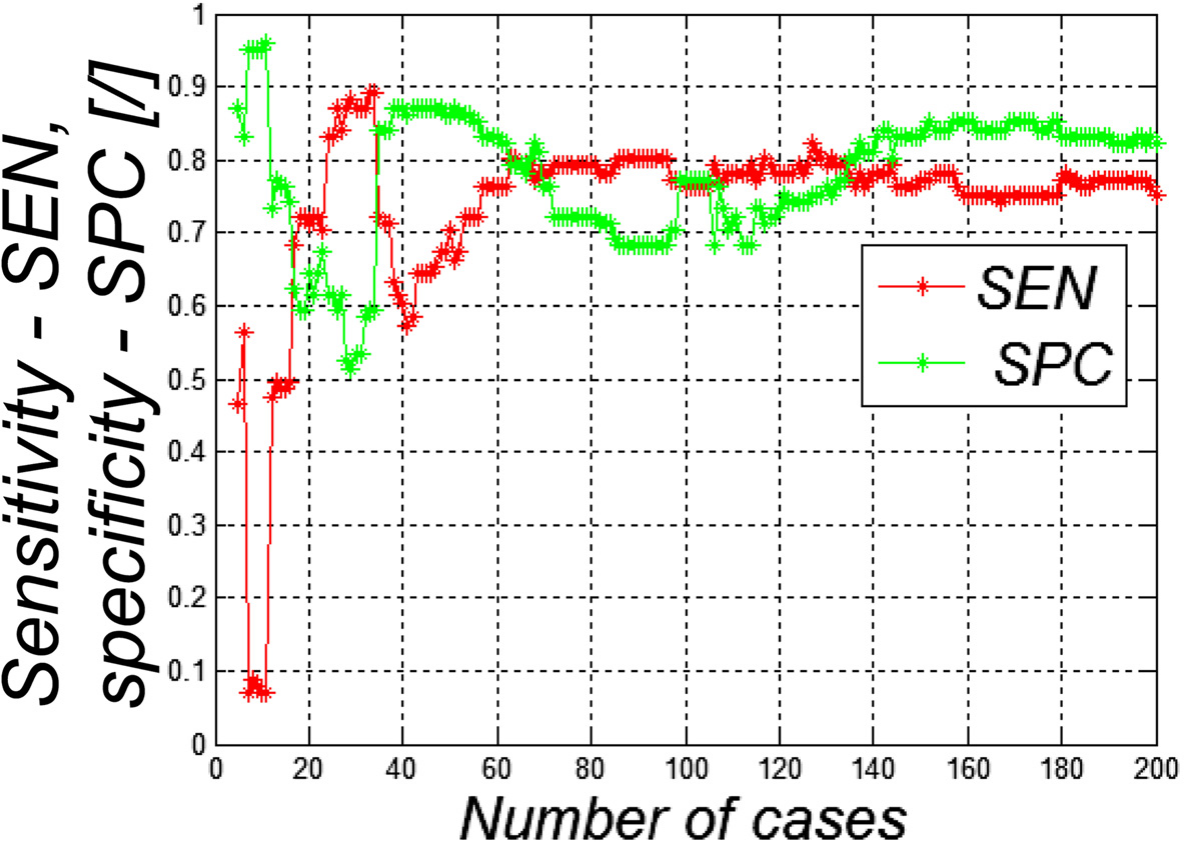
**Sensitivity ( *SEN *) and specificity ( *SPC *) of classifier applied to a validation set of 50 healthy and 50 ill subjects.** The training set consisted of the indicated number of individuals with Hashimoto’s disease (horizontal axis) with an equal number of healthy subjects. The test set consisted of different individuals than those used for the training set. Ten attributes were defined for each image.

The sensitivity *SEN* and specificity *SPC* of the classifier as a function of the number of Hashimoto’s patients in the training set are shown in Figure 2. The sensitivity measures the proportion of true positives that are correctly identified by the classifier and is defined as *SEN* = *TP*/(*TP* + *FN*), while the specificity measures the proportion of true negatives that are correctly identified and is defined as *SPC* = *TN*/(*FP* + *TN*) where *TP* - true positive, *TN*- true negative, *FN*- false negative, *FP* - false positive. The accuracy *ACC* of the classifier is defined as the fraction of cases that are classified correctly, i.e. the true positive plus the true negative detection rates.As shown in Figure 2, the performance of the classifier is highly variable for training sets with fewer than about 60 cases (plus an equal number of healthy controls). For larger training sets the performance of the classifier approaches a sensitivity and specificity of 75-85%, which may possibly be useful for medical purposes. In this case the validation was statistically correct (the validation and training sets used images from different individuals) but the classifier performed poorly when trained with fewer than about 100 images. Readers should note the large size of the training and evaluation sets that are needed to train a useful classifier and to gain a reasonable understanding of its performance.

b. Effect of different operators and equipment.

We next consider the performance of the classifier using images acquired by two operators with different ultrasound apparatus in different medical units. Both operators 1 and 2 use both medical units 1 and 2. The classifier was created with a training set of 50 healthy subjects and 50 ill patients obtained only by the first operator, and applied to independent validation sets of the same size acquired by either operator (Table 1). The classifier generally performed better for Operator 1 (who had acquired the data) than for Operator 2.

**Table 1:** Impact of the operator and the device type on the sensitivity and specificity of the classifier SVM

**Operator\device**	**1**	**2**
1	*SEN* = 75%	*SEN* = 68%
	*SPC* = 82%	*SPC* = 61%
2	*SEN* = 68%	*SEN* = 58%
	*SPC* = 59%	*SPC* = 70%

In this case, the SVM had been trained on 100 images (50 healthy, 50 ill patients) acquired by Operator 1 and validated using independent sets of the same size of images obtained by Operator 1 or 2. The performance of the classifier is generally better with Operator 1 on data collected on device 1 (which originated the data) although there are variations in performance. Both operators used both devices to acquire data.

c. Assessment of attribute relevance

We now consider how the selection of attributes affects the performance of the classifier using the previously described images. The input set of 10 attributes was divided into subgroups containing all their combinations – a total of 1023 SVM classifiers. The classifier was trained with the same set of 400 individuals as before (half ill, half healthy) using combinations of attributes shown in Table 2, and tested with a holdout group of 100 different individuals (half healthy, half ill). The sensitivity (*SEN*), specificity (*SPC*) and accuracy (*ACC*) of the classification is shown in Table 2. This process had been conducted using four different kernel functions available in Matlab’s Statistics Toolbox; Table 2 shows the results obtained for the quadratic kernel basis.

**Table 2:** Results of training a classifier with SVM (quadratic kernel), using a training set of 400 ultrasound images (half from healthy individuals, half from individuals with Hashimoto’s disease) with a validation set of 100 separate individuals, half of whom had the disease

**Attribute number from 1 to 10**										***SEN***	***SPC***	***ACC***
**(1 - occurs, 0 – does not occur)**												
**1**	**2**	**3**	**4**	**5**	**6**	**7**	**8**	**9**	**10**			
1	0	1	0	1	0	0	0	0	0	0.831	0.792	0.811
1	1	1	0	1	1	0	1	0	0	0.792	0.831	0.811
1	1	1	0	1	0	1	1	0	0	0.772	0.851	0.811
1	1	1	0	1	0	0	0	0	0	0.831	0.782	0.806
0	1	0	1	1	0	0	0	0	0	0.772	0.841	0.806
1	1	0	1	1	0	0	0	0	0	0.782	0.831	0.806
…												
1	1	1	1	1	1	1	1	1	1	0.732	0.811	0.772
…												

*SEN, SPC, and ACC* refer to the sensitivity, specificity, and accuracy of the classifier with the validation set. The accuracy of the classifier does not increase significantly when more attributes are added, implying that some of the attributes contribute little to the performance of the classifier. The table shows the performance of the classifier when constructed from combinations of attributes (attributes used are indicated by 1, not used by 0).

Notably, as shown in Table 2, in some cases the classifier performed *better* when it had been trained using fewer attributes; it seems that only 3–4 of the attributes contributed significantly to the classification. Additional attributes may have reduced the performance of the classifier due to overfitting. Reducing the number of attributes greatly speeds up the process of classification and may reduce the required sizes of the training sets to avoid overfitting. Additionally, it simplifies the medical interpretation of the process, by directing attention to the most important attributes. However, choosing attributes in this retrospective manner introduces a post-hoc element into the analysis that, at least, needs to be acknowledged by the investigators and, ideally, should be followed up by subsequent studies in which the choice of attributes had been settled on *a priori*.These cases illustrate the extreme difficulty of developing effective classifiers from relatively small training sets, and the ease with which an investigator can be mislead. First, lack of independence of the training and validation sets can bias the validation tests, and the problem is worse with smaller datasets. Figure 1 shows an extreme case where the training and validation sets are the same. However, “independence” is not a binary quantity. Hidden correlations between the training and validation sets can lead to over optimism about the performance of the classifier. Second (Figure 2), classifiers that are developed from too-small training sets are likely to generalize poorly.

The bottom line: developing useful classifiers can require much larger data sets than are typically used in most biomedical engineering studies.

### Recommendations

The following recommendations are offered to investigators and readers/paper reviewers on the use of machine learning techniques in biomedical engineering research.

1. Authors clearly state the purpose and intended applications of their work.

The work may be viewed as exploratory data analysis, where the research is still trying to identify relationships among variables. In that case the authors should emphasize in their discussion that they were performing exploratory analysis and that confirmatory data analysis is required. On the other hand, if the goal of the study is to develop a predictive model, particularly one for medical diagnosis, then there must be either a physiological basis for the model or the investigators must have performed an appropriate analysis to establish its predictive value. Developing and validating a medically useful diagnostic test would require far more than exploratory classifier studies, and investigators should not over-promise what their studies can deliver.

2. Investigators should minimize the number of attributes in their classifiers.

This is a particularly urgent need with small studies, which are prone to overfitting with even a small number of attributes. Several approaches can be recommended to achieve this. One is to remove one variable at a time, run the classifier, and determine whether the sensitivity and specificity improve or degrade (for an example see one of the cases discussed above). Another approach is to use decision trees to pare down the variables and then process the remaining set with the classifier. Another is to use the method proposed by Weigand [19] in the context of neural networks, which involves use of a weight elimination algorithm to pare down the number of variables. These methods if used properly can avoid the bias introduced when authors select the variables to be used in training a classifier.

3. Investigators should address the issue of transportability.

How can a researcher at a different institution apply the work? Must he or she collect another full set of training data and train a SVM de novo? The authors, as experts on the particular methodology developed, should provide some opinion as to what portions of the method can be reused directly, and which might require “tuning” for implementation elsewhere. A new diagnostic technique is of no value to medicine unless other experts can apply it.

4. Investigators should view building classifiers not simply as an add-on statistical analysis, but as part and parcel of the experimental process, with much of that experiment being performed computationally.

As in physical experiments, the authors should provide sufficient information to allow others to repeat the numerical experiment.

In particular, if the goal of developing a classifier is to develop a medical diagnostic technique, the investigator is beginning a long process that would be needed to establish the sensitivity and specificity of the technique with the entire relevant patient population. Investigators can increase their chances of success by choosing attributes in collaboration with physicians in relevant fields, and performing a sensitivity analysis at an early stage to reduce the number of attributes needed.

Moskowitz et al. [20] provided guidelines for early stage development of diagnostic imaging applications, which apply to developers of classifiers as well. These authors pointed out that in early stages of development one cannot prove that a diagnostic method “works” in any medically useful way, but only that it cannot work, if that is the case, and it is better to uncover such bad news early rather than late in the development process. That requires preliminary studies with elements of good design include proper blinding and avoiding changing evaluation criteria during the tests. These considerations extend far beyond the use of packaged statistics software and learning classifiers.

## Competing interests

The authors declare that they have no competing interests.

## Authors’ contributions

KRF took the lead in writing, RK conducted numerical simulations and contributed to the writing, JDS provided advice, technical suggestions, and contributed text to the final version. All authors read and approved the final manuscript.
